# Reproducibility and reliability of flow quantification using CMR 2D-phase contrast and 4D-Flow in secondary mitral valve regurgitation

**DOI:** 10.1007/s10554-025-03421-x

**Published:** 2025-05-16

**Authors:** Yasaman Safarkhanlo, Martina Boscolo Berto, Giancarlo Spano, Benedikt Bernhard, Jonathan Schütze, Anselm W. Stark, Fabien Praz, Isaac Shiri, Alan A. Peters, Christof Schaub, Eva S. Peper, Chrysoula Garefa, Andreas Wahl, Jessica A. M. Bastiaansen, Christoph Gräni

**Affiliations:** 1 Department of Cardiology, Inselspital, University Hospital Bern, Bern, CH-3010 Switzerland; 2 Graduate School for Cellular and Biomedical Sciences (GCB), University of Bern, Bern, Switzerland; 3 Translation Imaging Center (TIC), Swiss Institute for Translational and Entrepreneurial Medicine, Bern, Switzerland; 4 Department of Diagnostic, Interventional and Pediatric Radiology (DIPR), Inselspital, University Hospital Bern, Bern, Switzerland

**Keywords:** Cardiovascular, Data analysis, Challenges, Flow, Heart, Valves, Velocity & flow, Validation, 4D-flow, 2D-PC

## Abstract

Accurate quantification of mitral valve regurgitation (MVR) is crucial for patient management. While different MVR quantification methods based on cardiac magnetic resonance imaging (CMR) exist, their reproducibility and reliability remain uncertain. This study aims to evaluate the reproducibility of different CMR 2D-phase contrast (PC) and 4D-flow MVR quantification methods. The inter-reader and intra-reader reproducibility were assessed using intraclass correlation coefficients (ICC). Seven methods were evaluated: 2D-PC standard (LVSV minus aortic flow), 2D-PC mitral-aortic (mitral inflow minus aortic flow), 2D-PC direct (quantifying mitral backflow), 4D-flow standard, 4D-flow mitral-aortic, 4D-flow direct, and volumetric method (LVSV minus RVSV) in 32 patients (74.8 ± 9.8 years, 28% females) with secondary MVR, analyzed independently by two experienced readers. A total of 26 patients were included in the analysis for 2D-PC and 15 for 4D-flow methods. Among all techniques, 2D-PC standard was the most reliable method with both good inter-reader (ICC = 0.85, *p* < 0.001) and intra-reader agreement (ICC = 0.87, *p* < 0.001). The 4D-flow standard (ICC = 0.97, *p* < 0.001) and the volumetric method (ICC = 0.81, *p* < 0.001) showed excellent and good intra-reader agreements, respectively, but only moderate inter-reader reproducibility (ICC = 0.52, *p* = 0.027 and ICC = 0.71, *p* < 0.001). In patients with secondary MVR, 2D-PC standard method demonstrated the highest reproducibility, while 4D-flow methods showed excellent intra-reader reliability but more variable inter-reader agreement. Standardized post-processing protocols and training would likely enhance the clinical application of these techniques. Future studies should investigate these methods in larger, diverse cohorts and correlate findings with clinical outcomes.

**Supplementary Information:**

The online version contains supplementary material available at 10.1007/s10554-025-03421-x.

## Introduction

Secondary mitral valve regurgitation (MVR) is a clinically significant condition that arises from structural and functional changes in the left ventricle and left atrium, often associated with chronic heart failure. Unlike primary MVR, which is defined by the presence of pathologies of the mitral valve apparatus, secondary MVR is driven by mitral annular dilation and tethering of the mitral leaflets. These changes frequently lead to the displacement of the papillary muscles and chordae tendineae, resulting in impaired leaflet coaptation and subsequent regurgitation of left ventricular stroke volume into the left atrium. The evaluation of secondary MVR is challenging due to its heterogeneous clinical presentation, complex leaflet geometry, and its dynamic nature, which often varies with loading conditions and ventricular function [1, 2]. With the wide availability of surgical and transcatheter edge-to-edge repair (TEER) treatment options, accurate quantification of MVR is crucial for effective risk stratification and treatment planning [3, 4]. Current international practice guidelines recommend treatment of severe secondary MVR based on the assessment of regurgitant volume and fraction [5, 6].

In clinical practice, transthoracic and transesophageal echocardiography remain the primary imaging modalities for assessing MVR. They allow for qualitative evaluations of valve morphology and regurgitant jet characteristics as well as semi-quantitative and quantitative measurements such as vena contracta width and regurgitant volume. However, echocardiographic assessment can be limited by user dependency and suboptimal echocardiographic window, which may lead to variability and bias [2, 7].

Ongoing advances have highlighted cardiac magnetic resonance imaging (CMR) as a promising alternative for accurate and reproducible quantification of MV regurgitant volume. Recent studies [8, 9] have shown that while echocardiography provides superior spatial resolution compared to CMR, CMR offers distinct advantages in volumetric quantification and tissue characterization through its three-dimensional assessment capabilities. These strengths can enhance the accuracy of regurgitant volume measurement and aid in risk stratification. However, while there are multiple CMR methods to quantify MVR, no consensus exists on a preferred method, and comprehensive data comparing the performance and reliability of traditional 2D-phase contrast (PC) methods and emerging 4D-flow techniques remain limited. The present study aims to systematically evaluate and compare multiple established approaches, including volumetric, 2D-PC, and 4D-flow quantification methods for secondary MVR, focusing on their reproducibility and reliability through inter-method, inter-reader, and intra-reader agreements.

## Methods

### Study population

Consecutive patients with secondary MVR and reduced left ventricular ejection fraction (EF < 50%) evaluated for TEER between September 2021 and September 2024 underwent CMR. Patients were recruited prospectively to the *Prediction of Reverse Remodeling and Outcome in Patients With Severe Secondary Mitral Valve Regurgitation Undergoing Transcatheter Edge-to-edge Mitral Valve Repair* (PRE-MITRA) study (NCT04913727) after written informed consent. Exclusion criteria were age below 18 years, pregnancy or breastfeeding, severely impaired renal function (GFR < 15 ml/min), untreated severe concomitant valve diseases, such as severe tricuspid valve regurgitation, severe claustrophobia, or any other contraindication for CMR. The study was approved by the local ethics committees (KEK-BE 2021 − 00704) and was conducted following the Declaration of Helsinki.

### CMR data acquisition and measurements

CMR scans were performed on 1.5T (MAGNETOM Sola, Siemens Healthcare, Erlangen, Germany) (*N* = 28) and 3T (MAGNETOM Prisma, Siemens Healthcare, Erlangen, Germany) (*N* = 4) scanners. Cine steady-state free precession (bSSFP) sequences were employed with retrospective electrocardiographic (ECG) gating with breath-hold. Standard cardiac geometries were acquired, encompassing multiple short-axis slices with no interslice gaps, covering the entire left ventricle (LV) and long-axis two-, three-, and four-chambered views. The reconstructed in-plane spatial resolution was 2.1 × 2.1 mm^2^, with 8.0 mm slice thickness and 25 cardiac phases.

Additionally, different 2D-PC flow measurements were conducted: one in the ascending aorta, with the imaging plane positioned 10 mm above the aortic valve and perpendicular to the aortic flow direction, and the other across the mitral area valve. Velocity encoding (VENC) was set typically to 150 cm/s, adjusted individually if necessary. ECG gating was used for both acquisitions, with an in-plane spatial resolution of 2.0 × 2.0 mm^2^, temporal resolution of 25 phases per cardiac cycle, and slice thickness of 7 mm.

Out of the 32 patients enrolled in this study, only 15 were able to undergo additional 4D-flow acquisition. The remaining patients were excluded from this protocol extension due to their inability to tolerate prolonged scanning times, attributed to frailty, reduced compliance, or limitations in maintaining a stable position for the extended acquisition duration. For these patients, the 4D-flow data were acquired with a spatial resolution of 2.5 × 2.5 × 2.5 mm^3^ and a temporal resolution of 25 phases per cardiac cycle. VENC was set to 120 cm/s, with adjustments made as necessary to optimize data quality. All acquisition parameters are summarized in Table 1.

**Table 1:** Acquisition parameters

Acquisition parameters	Spatial resolution (mm^3^)	Cardiac phases#	TE/TR (ms)	Segments#
Short axis cine	2.1 × 2.1 × 8.0	25	1.07/2.55	19
2D-flow	2.0 × 2.0 × 6.0	25	2.37/4.23	2
4D-flow	2.5 × 2.5 × 2.5	25	2.35/4.68	6

### Image analysis and flow quantification methods

Image analysis was performed using CVI42 software (Circle Cardiovascular Imaging, Calgary, Canada, version 6.0.2) with two readers (authors YS and MB) blinded to the patient’s clinical characteristics and outcomes. Measurements were performed twice by the same reader (A1 and A2) and once by a different reader (B). Outliers with Z-scores greater than two were excluded to ensure data clarity and consistency.

**Fig. 1:**
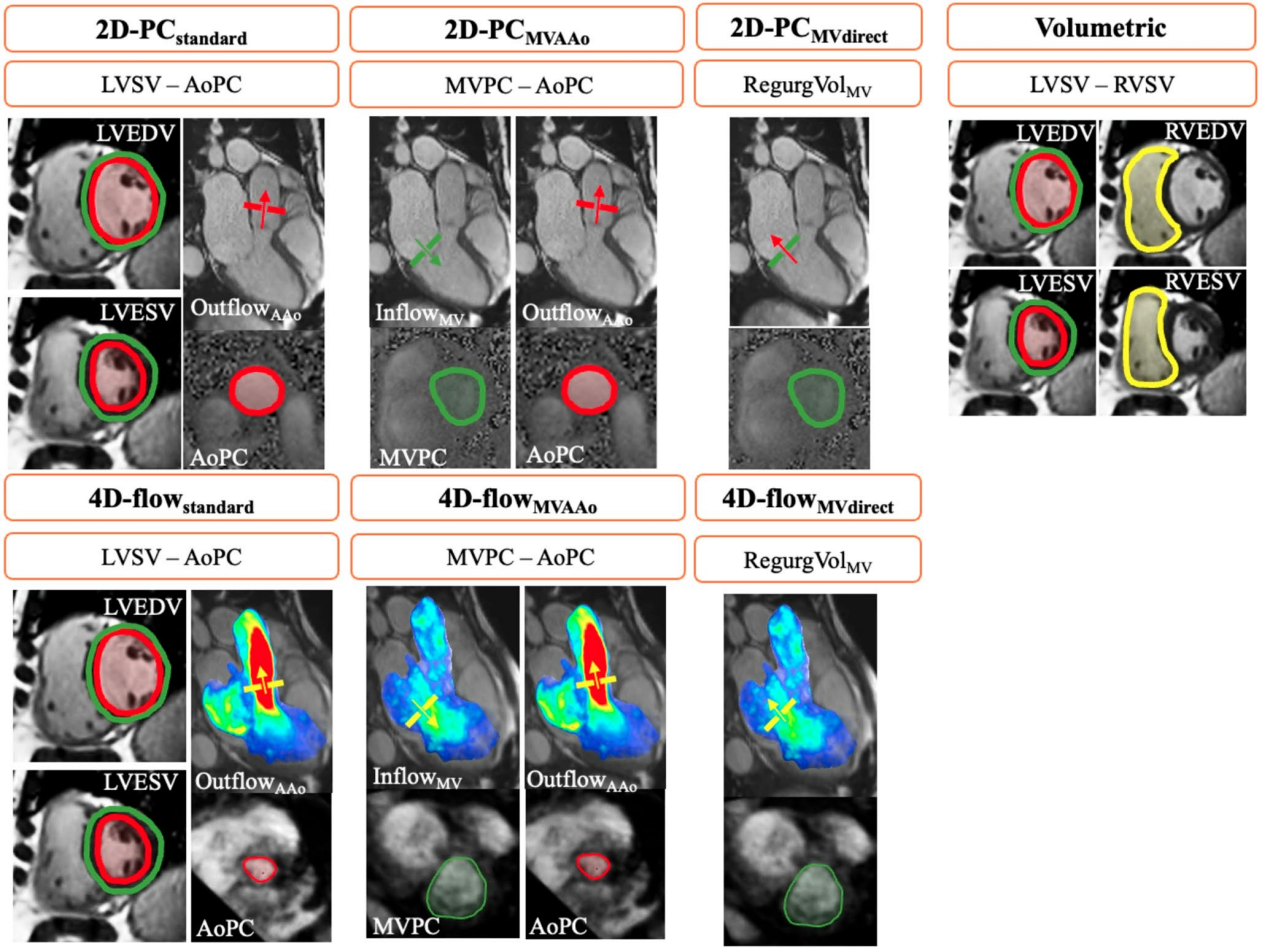
Illustration of MVR quantification methods. 2D-PC_standard_, CMR flow gold standard (Left Ventricle Stroke Volume [LV SV]– Aortic Forward Flow Volume derived from 2D-PC plane [AoPC]); 2D-PC_MVAAo_, Forward Flow through Mitral Valve (MVPC) - AoPC; 2D-PC_MVdirect_, directly quantifying flow through Mitral Valve (Backward flow through Mitral Valve [RegurgVol_MV_]); Volumetric (LV SV– Right Ventricle Stroke Volume [RV SV]); 4D-flow_standard_, similar to 2D-PC_standard_ using 4D-flow sequence instead of 2D-PC (LV SV– AoPC); 4D-flow_MVAAo_, similar to 2D-PC_MVAAo_ using 4D-flow sequence instead of 2D-PC (MVPC– AoPC); 4D-flow_MVdirect_, directly quantifying flow through Mitral Valve (Backward flow through Mitral Valve [RegurgVol_MV_]); AoPC, Aortic Forward Flow (Outflow_AAo_); MVPC, Mitral Valve Forward Flow (Inflow_MV_); EDV, End Diastolic Volume; ESV, End Systolic Volume

Seven distinct methods for quantifying MVR volume using CMR were employed in this study (Fig. 1): (1) 2D-PC standard method (2D-PC_standard_), which indirectly calculates MVR volume by subtracting the 2D-PC measured aortic outflow volume (AoPC), from the left ventricular stroke volume (LVSV) volumetrically obtained from standard 2D cine short-axis CMR images; (2) 2D-PC mitral valve method (2D-PC_MVAAo_), quantifying MVR volume by subtracting AoPC from the forward flow through the mitral valve (MVPC), both measured using 2D-PC planes; (3) 2D-PC mitral valve direct method (2D-PC_MVdirect_), directly measuring the regurgitation volume from the 2D-PC plane on the mitral valve; (4) 4D-flow standard method (4D-flow_standard_ ), which indirectly calculates MVR volume by subtracting AoPC, measured using 4D-flow CMR, from LVSV derived from standard 2D cine short-axis CMR images; (5) 4D-flow mitral valve (4D-flow_MVAAo_), quantifying MVR volume by subtracting AoPC from MVPC, both assessed using 4D-flow CMR; (6) 4D-flow mitral valve direct method (4D-flow_MVdirect_), directly measuring the regurgitation volume from the flow plane on the mitral valve using 4D-flow acquisition data; and (7) the Volumetric method that calculates MVR volume as the difference between LVSV and right ventricular stroke volume (RVSV), both obtained from standard 2D cine CMR images.

**Fig. 2:**
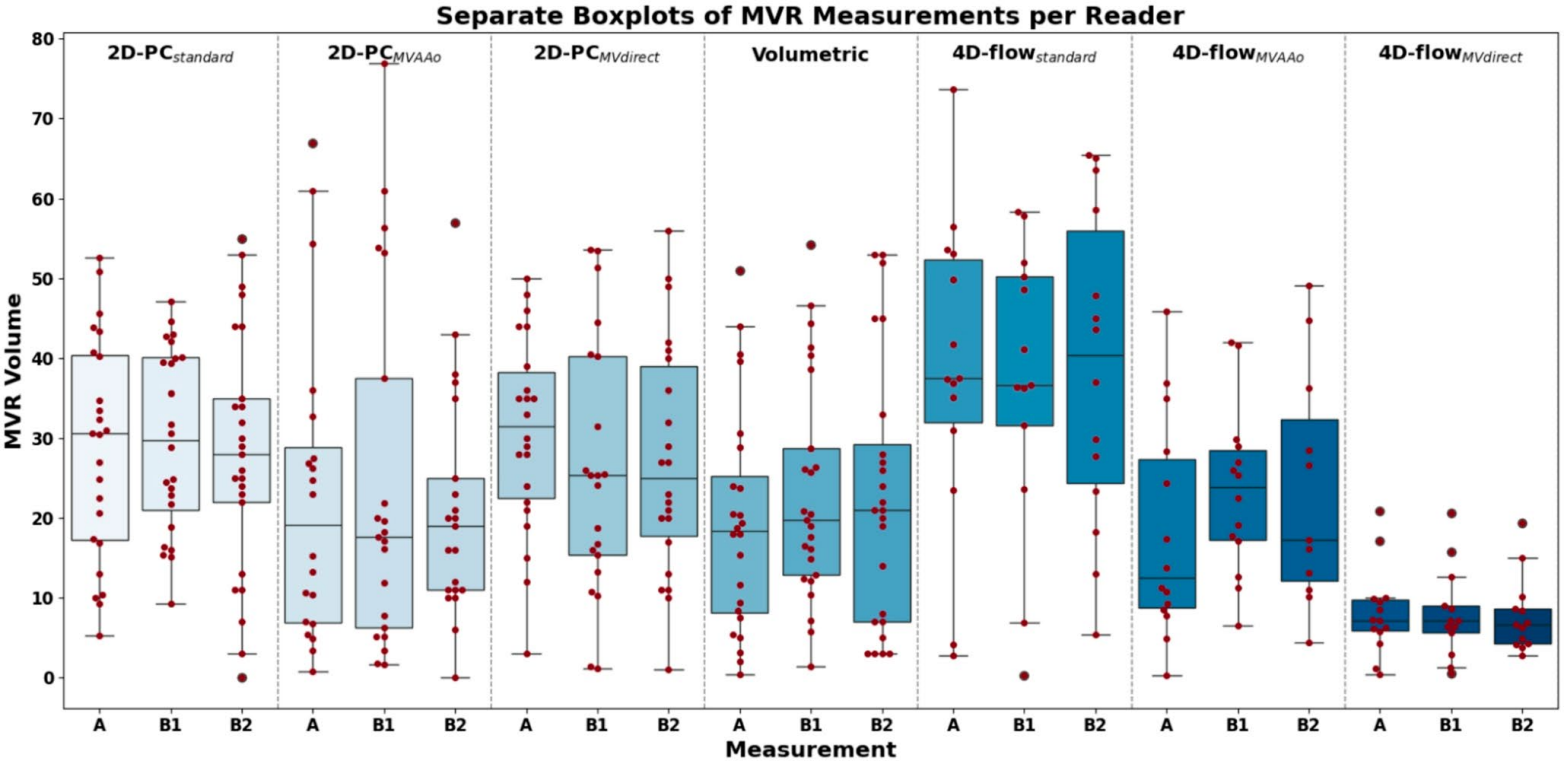
Box plots of Mitral Valve Regurgitation (MVR) volumes measured using various CMR flow quantification methods. Measurements were taken with seven distinct methods, based on a cohort comprising 26 patients with 2D-PC data, of whom 15 patients also underwent 4D-flow acquisition. Each method was analyzed by two readers: once by reader A and twice by the same reader (B1 and B2). Outliers (Z-score > 2) were removed from the data to enhance clarity. Each method is illustrated with its corresponding box plot, with individual measurement points overlayed in red

All 4D-flow measurements, including both aortic and mitral valve flow quantifications, were performed using valve tracking functionality in CVI42. For each valve, regions of interest (ROIs) were adapted throughout the cardiac cycle to account for in-plane motion and vessel dilation. For the direct mitral regurgitation measurements, the analysis plane was placed at the level of the mitral valve annulus, consistent with the corresponding 2D-PC acquisition planes, ensuring cross-method consistency. The regurgitant jet was also tracked throughout the cardiac cycle using the retrospective valve tracking feature to ensure accurate capture of flow dynamics across all time points.

### Statistical analysis

Inter- and intra-reader agreement analyses were performed to evaluate the consistency and reliability of MVR quantification methods by calculating intraclass correlation coefficients (ICC). ICC values less than 0.50 indicate poor agreement, 0.50 to 0.75 indicate moderate agreement, 0.75 to 0.90 indicate good agreement, and values greater than 0.90 indicate excellent agreement [10, 11].

Bland-Altman plots were generated to visually assess the agreement between different measurements for inter- and intra-reader analyses, highlighting any systematic biases or discrepancies between these measurements.

MVR severities were classified into three categories based on the quantified MVR volume, following established clinical thresholds [12]: mild (MVR volume less than 30 ml), moderate (MVR volume between 30 and 59 ml), and severe (MVR volume greater than 60 ml).

Statistical analyses were conducted using Python (version 3.9.6). Statistical significance was denoted using stars: * indicates *p* < 0.05, ** indicates *p* < 0.01, and *** indicates *p* < 0.001.

## Results

A total of 32 patients (74.8 ± 9.8 years, 28% females) underwent CMR-based MVR quantification and enrolled in the study (Fig. 2). Of these, six patients were excluded from the 2D-PC analysis due to poor image quality caused by atrial fibrillation. A total of 26 underwent 2D-PC analysis and 15 patients underwent additional 4D-flow methods (i.e. underwent all flow quantification methods). Out of these, 11 had atrial fibrillation, but the image quality was adequate for analysis. Patient demographics are summarized in Table 2.

**Table 2:** Patient demographics. Summary of patient demographics for all patients, 2D-PC acquisition, and 4D-flow acquisition. Each column presents data as mean ± standard deviation (std), with the range indicated in parentheses

Patient demographics	All patients	2D-PC acquisition	4D-flow acquisition
Number (male: female)	32 (25: 7)	26 (21: 5)	15 (11: 4)
Age (years)	75 ± 10 (49–88)	74 ± 10 (49–88)	75 ± 9 (56–88)
Weight (kgs)	80 ± 25 (48–170)	79 ± 23 (52–170)	79 ± 29 (52–170)
Height (cm)	173 ± 9 (155–195)	172 ± 9 (155–195)	174 ± 11 (155–195)
BMI (kg/m^2^)	26 ± 6 (18–45)	26 ± 6 (18–45)	26 ± 7 (18–45)
Heart rate (bpm)	77 ± 17 (33–105)	74 ± 16 (33–105)	77 ± 18 (33–105)
CMR parameters			
LVEF (%)	32 ± 12 (13–67)	32 ± 12 (13–67)	37 ± 9 (22–51)
LVEDV (ml)	283 ± 105 (110–471)	288 ± 104 (110–471)	241 ± 66 (171–333)
RVEF (%)	42 ± 15 (19–67)	42 ± 15 (19–67)	50 ± 17 (25–64)
RVEDV (ml)	162 ± 47 (55–288)	155 ± 42 (55–221)	151 ± 51 (55–208)

### Inter- and intra-reader agreements

**Fig. 3:**
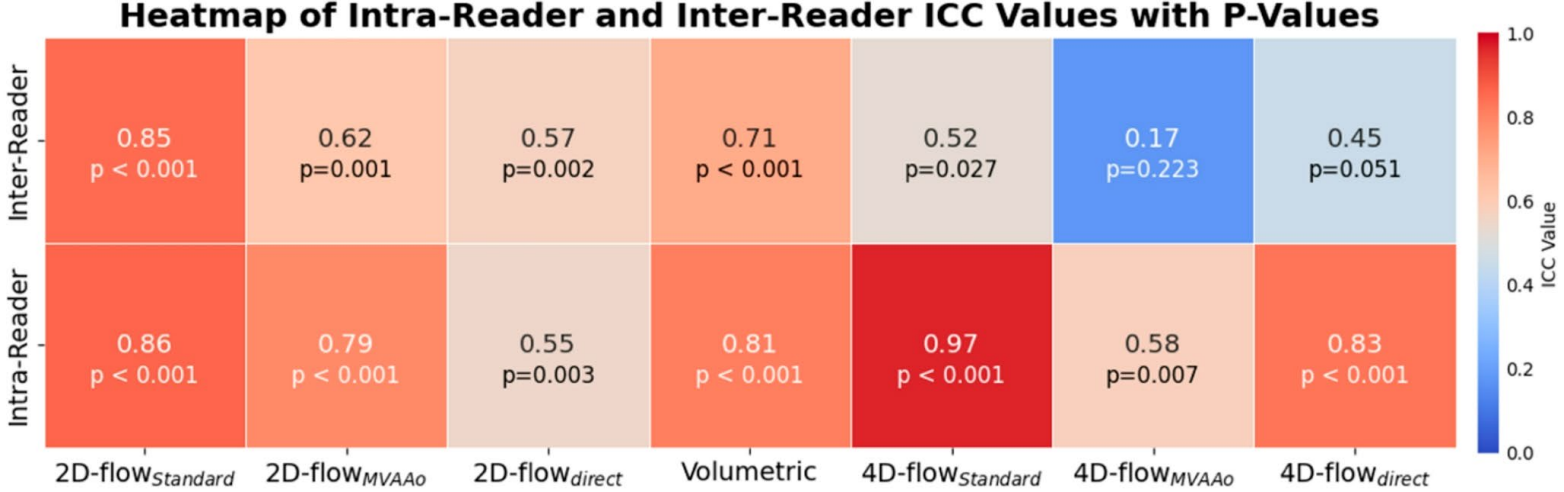
Heatmap of Intra-Reader and Inter-Reader Intraclass Correlation Coefficient (ICC) values for MVR volume measurements across various CMR flow quantification methods. The analysis includes the same 15 patients who underwent both 2D-PC and 4D-flow acquisitions. Each cell displays the ICC value, with p-values annotated below. Methods include both 2D and 4D-flow measurements, as well as Volumetric analysis, comparing reliability between repeated measurements by the same reader (Intra-Reader) and measurements by different readers (Inter-Reader). The color scale represents ICC values, with darker red colors indicating stronger reliability and darker blue colors indicating weaker reliability. (ICC > = 0.9, excellent reliability; ICC = 0.75–0.9, good reliability; ICC = 0.5–0.75, moderate reliability; ICC < 0.5, poor reliability.)

The results for inter- and intra-reader agreements are summarized in Fig. 3. The 2D-PC_standard_ showed good inter-reader reliability (ICC = 0.851, 95% CI: 0.70 to 0.93, *p* < 0.001) and intra-reader reliability (ICC = 0.865, 95% CI: 0.72 to 0.94, *p* < 0.001), indicating good consistency between measurements. Conversely, the 2D- PC_MVAAo_ exhibited moderate inter-reader reliability (ICC = 0.618, 95% CI: 0.28 to 0.82, *p* = 0.001) and intra-reader reliability (ICC = 0.791, 95% CI: 0.56 to 0.91, *p* < 0.001). The 2D-PC_MVdirect_ also demonstrated moderate inter-reader reliability (ICC = 0.574, 95% CI: 0.22 to 0.80, *p* = 0.002) and intra-reader reliability (ICC = 0.547, 95% CI: 0.18 to 0.78, *p* = 0.003). The 4D-flow_standard_ demonstrated moderate inter-reader reliability (ICC = 0.524, 95% CI: -0.01 to 0.82, *p* = 0.027) and excellent intra-reader reliability (ICC = 0.966, 95% CI: 0.90 to 0.99, *p* < 0.001). The 4D-flow_MVAAo_ showed weak inter-reader agreement (ICC = 0.173, 95% CI: -0.23 to 0.59, *p* = 0.223) and moderate intra-reader reliability (ICC = 0.579, 95% CI: 0.12 to 0.84, *p* = 0.007). The 4D- flow_MVdirect_ showed poor inter-reader reliability (ICC = 0.450, 95% CI: -0.10 to 0.78, *p* = 0.051) and good intra-reader reliability (ICC = 0.833, 95% CI: 0.57 to 0.94, *p* < 0.001). The volumetric method demonstrated moderate reliability, with inter-reader ICC of 0.708 (95% CI: 0.45 to 0.86, *p* < 0.001) and intra-reader ICC of 0.814 (95% CI: 0.63 to 0.91, *p* < 0.001).

**Fig. 4:**
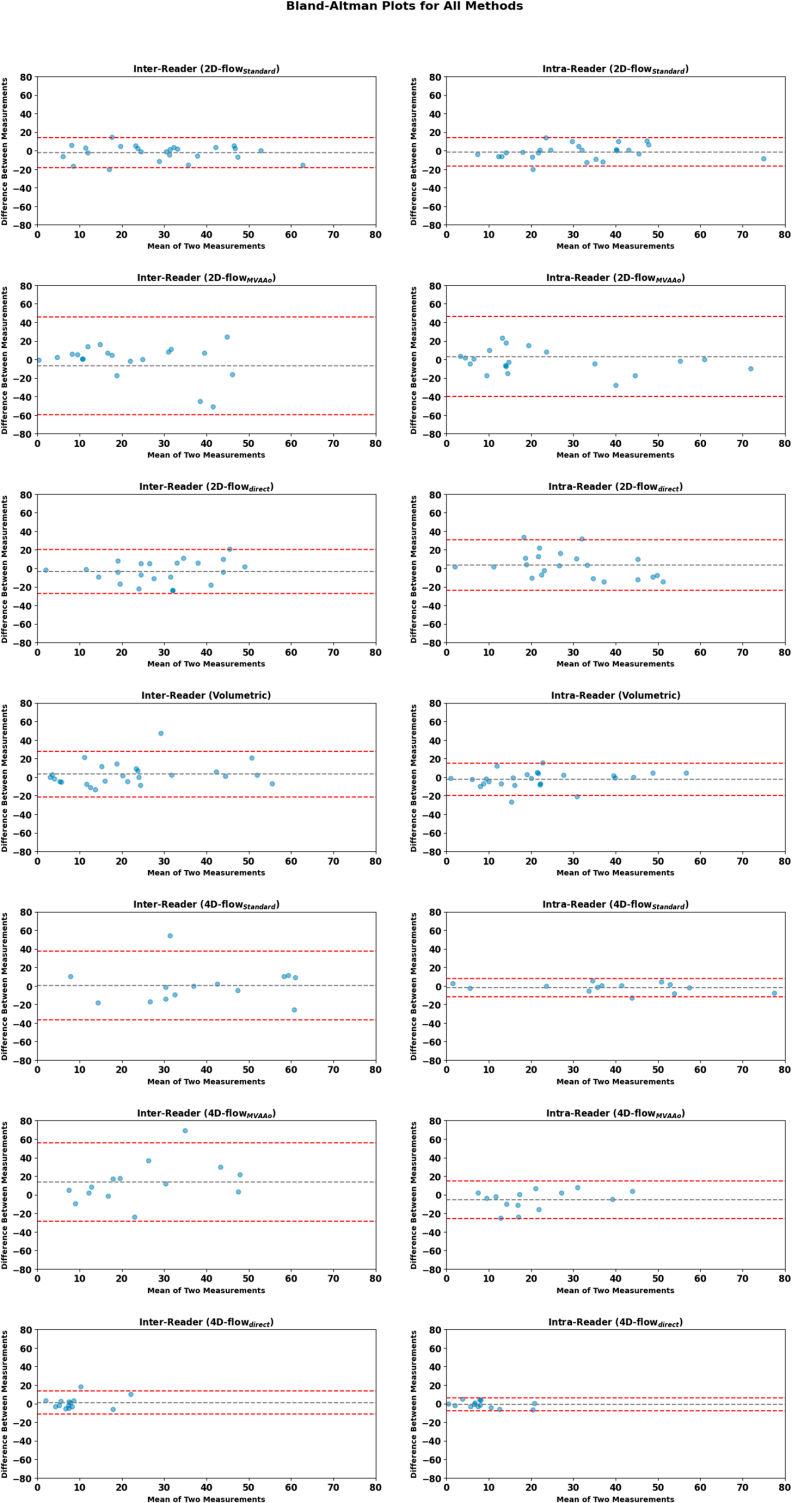
Bland-Altman plots comparing inter- and intra-reader measurements of MVR volumes across various CMR flow quantification methods. The analysis includes 26 patients for 2D-PC methods and 15 patients for 4D-flow methods. Each plot displays the mean of two measurements on the x-axis and the difference between them on the y-axis, with limits set from 0 to 80 on the x-axis and − 80 to + 80 on the y-axis. Horizontal dashed lines indicate the mean difference (gray) and the limits of agreement (± 1.96 SD, red). Inter-reader plots (comparing two different readers) are shown first for each method, followed by intra-reader plots (comparing repeated measurements by the same reader), providing insights into the consistency of measurements across and within readers for each quantification method

Overall, the methods exhibiting the highest consistency across both readers and repeated measurements were 2D-PC_standard_, 4D-flow_standard_, and the volumetric method, while the 2D-PC_MVdirect_ and 4D-flow_MVAAo_ methods demonstrated greater variability in agreement (Fig. 4).

### MVR severity classifications across methods

**Fig. 5:**
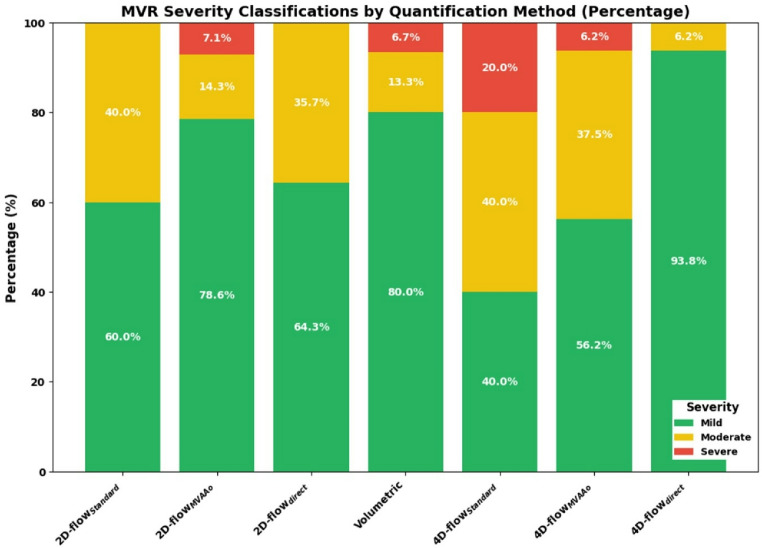
MVR severity classification of mitral valve regurgitation (MVR) across various CMR flow quantification methods. The stacked bar chart shows the distribution of a subset of cases, the patients with both 2D-PC and 4D-flow acquisitions (*n* = 15), classified as Mild, Moderate, or Severe for each method. Percentages within the bars indicate the relative contribution of each severity category, providing a detailed comparison of the classification outcomes for different measurement techniques

The MVR severity classification results were calculated for all patients, with missing values considered for each method. The results for each method are as follows (Fig. 5):

For the patients with 2D-PC acquisition, the 2D-PC_standard_ method classified the majority of cases as mild (60%), followed by moderate (40%), with no cases classified as severe. The 2D-PC_MVAAo_ classified 79% of cases as mild, 14% as moderate, and 7% as severe. Similarly, the 2D-PC_MVdirect_ showed 64% of cases classified as mild, and 36% as moderate, with no cases classified as severe.

The volumetric method primarily classified cases as mild, with 80% of cases in this category, followed by 13% of cases as moderate and 7% as severe.

In contrast, for the 4D-flow acquisition, the 4D-flow_standard_ method classified 40% of cases as mild, 40% as moderate, and 20% as severe. The 4D-flow_MVAAo_ method classified 56% of cases as mild, 38% as moderate, and 6% as severe. The 4D-flow_MVdirect_ displayed a distribution with 94% of cases as mild, 6% as moderate, and none as severe.

## Discussion

This study evaluated various CMR methods for quantifying MVR volume assessing their reliability, reproducibility, and agreement in patients with secondary MVR evaluated for TEER. Among the tested methods, 2D-PC_standard_ demonstrated the highest reliability in both inter- and intra-reader agreements, while 4D-flow_standard_ exhibited excellent intra-reader reliability but moderate inter-reader reliability. It is important to note the distinct workflows between 2D-PC and 4D-flow acquisitions in this study. For 2D-PC methods, imaging planes were selected during scan acquisition, with both readers subsequently analyzing these identical pre-determined planes. In contrast, 4D-flow acquisitions allowed readers to retrospectively select and position analysis planes during post-processing. This methodological difference reflects the actual clinical workflow of these techniques.

The observed high reproducibility of the 2D-PC_standard_, 4D-flow_standard_, and volumetric methods is consistent with findings from other studies [13–18] and supports their use in clinical practice where reproducibility is crucial. Specifically, the high intra-reader ICC for the 4D-flow_standard_ method highlights its capability to provide accurate and consistent measurements when analyzed by the same reader. The disagreement observed in inter-reader reliability emphasizes the need for standardized post-processing protocols and training for a more consistent application of this method in clinical practice. On the other hand, the moderate inter-reader reliability observed for 2D-PC_MVdirect_ and 4D-flow_MVAAo_ may indicate that these methods are influenced by subjective elements in interpretation and variations in reader technique. This may include placing the flow plane or contouring the valve, related to the mitral valve’s saddle shape and through-plane motion [19]. The eccentricity of regurgitant jets, a hallmark of secondary MVR [20], further compounds these challenges. Eccentric jets often interact with surrounding structures and produce complex flow dynamics, which can lead to inaccuracies in both 2D-PC and 4D-flow quantifications. In 2D-PC imaging, the dependence on imaging plane orientation and velocity encoding makes it particularly vulnerable to underestimation or overestimation of regurgitant volumes. Our findings suggest that methods such as 2D-PC_standard_ and 4D-flow_standard_ may be more robust against these challenges. Particularly, 4D-flow_standard_ might offer distinct advantages in complex cases such as multi-valve regurgitation or eccentric jets [21], but further refinement and standardization of imaging protocols are needed.

In the present study, the classification of the severity of MVR showed significant inter-method variations. Surgical or transcatheter MVR treatment is recommended in severe MVR only, and varying severity grading highly complicates clinical decision-making. Our results demonstrate that the 2D-PC methods predominantly identify cases as mild or moderate, while 4D-flow methods and volumetric analysis exhibited broader distributions across severity categories. The higher proportion of severe cases if classified by 4D-flow techniques might be attributed to the more comprehensive assessment of regurgitant severity in patients with complex cardiac anatomy. 4D-flow can capture aspects of flow dynamics that other methods may overlook, such as multi-valve regurgitation, overlapping flow jets, or perpendicular flow plane orientation. As the only single-acquisition method that records intra-cardiac flow information across the same cardiac cycle, 4D-flow offers unique advantages for evaluating these complex hemodynamic patterns.

So far, the absence of a consensus on validated thresholds for CMR-derived MVR severity metrics, such as regurgitant volume and regurgitant fraction, limits comparability across imaging modalities and highlights the need for technique-specific reference values [21]. Standardized protocols for both acquisition and post-processing are crucial to improve inter-center reproducibility and ensure consistency in MVR quantification. This involves establishing consensus on key parameters, such as velocity encoding settings, pulse sequences, valve contouring techniques, and reconstructed plane placement in 4D-flow imaging [21]. Future studies should aim to evaluate a consistent cohort of patients across multi-modality quantification methods to define method-specific cutoff values for severe MVR.

### Limitations

This study has several limitations that warrant consideration. A key methodological limitation is the inherent difference in workflow between 2D-PC and 4D-flow quantification methods. With 2D-PC, imaging planes are prospectively selected during acquisition, creating fixed measurement planes that both readers subsequently analyze. In contrast, 4D-flow enables retrospective plane selection during post-processing, allowing each reader to independently position analysis planes. This fundamental difference likely contributed to the finding of higher inter-reader consistency for 2D-PC methods, as both readers analyzed identical pre-selected planes, rather than necessarily indicating intrinsic superiority of the technique itself. A truly direct comparison would require readers to independently select 2D-PC planes during acquisition, a design that was beyond the scope of this study.

The limited sample size and focus on patients with secondary MVR reduce the applicability of the results to those with milder forms or other forms, like primary MVR. Besides, only a subset of patients underwent 4D-flow acquisition due to the extended scan duration required for this protocol, which was not feasible for frailer patients or those with limited tolerance for prolonged imaging. This limitation not only reduced the statistical power for 4D-flow analyses but potentially introduced selection bias, as healthier patients may be overrepresented in the 4D-flow cohort.

Some patients included in this study also exhibited mild to moderate regurgitation in additional valves, such as tricuspid regurgitation. This concomitant valve dysfunction may have introduced variability across multiple quantification methods. The volumetric method, which relies on net flow calculations, is particularly prone to inaccuracies in such cases. 2D-PC techniques may also be affected due to overlapping regurgitant jets, while even advanced approaches like 4D-flow CMR can face challenges in accurately isolating and quantifying flow patterns from multiple valve lesions [8].

Additionally, around two-thirds of patients had atrial fibrillation, which can further impact measurements due to beat-to-beat variability in stroke volumes, irregular flow patterns, and difficulties in averaging data over the cardiac cycle. These factors can lead to reduced precision and reliability, particularly in methods sensitive to temporal variations in flow.

Finally, this study utilized CMR as a single imaging modality and did not compare multi-modal approaches incorporating echocardiography or invasive measurements, which might have provided additional context for interpreting the results.

## Conclusion

In patients with secondary MVR, 2D-PC_standard_ demonstrated the highest reproducibility with good inter- and intra-reader agreement, while 4D-flow_standard_ showed excellent intra-reader reliability but more variable inter-reader agreement. Standardized post-processing protocols and reader training would likely enhance the clinical application of 4D-flow techniques. The distinct workflows between 2D-PC methods (with pre-selected imaging planes) and 4D-flow techniques (allowing retrospective plane selection) affect reproducibility characteristics and should inform clinical implementation. Future studies should investigate these methods in larger, diverse cohorts and correlate reproducibility findings with clinical outcomes to optimize their application in clinical practice.

## Electronic supplementary material

Below is the link to the electronic supplementary material.

Supplementary Material 1

## Data Availability

No datasets were generated or analysed during the current study.

## Abbreviations

2D: Two-Dimensional
4D: Four-Dimensional
CI: Confidence Interval
CMR: Cardiovascular Magnetic Resonance
ECG: Electrocardiogram
EDV: End-Diastolic Volume
EF: Ejection Fraction
ESV: End-Systolic Volume
ICC: Intraclass Correlation Coefficient
LV: Left Ventricle
MVR: Mitral Valve Regurgitation
PC: Phase Contrast
RV: Right Ventricle
SD: Standard Deviation
SV: Stroke Volume
VENC: Velocity Encoding
