# Supplementary material for: Reproducibility and reliability of flow quantification using CMR 2D-phase contrast and 4D-Flow in secondary mitral valve regurgitation
Source: Int J Cardiovasc Imaging. 2025 May 16;41(7):1341–50. doi: 10.1007/s10554-025-03421-x (PMC12241284; doi:10.1007/s10554-025-03421-x)

**Supplementary Material**

**Table S1: Pearson's Correlation Coefficients and P-values**

| **Method Comparison** | **Pearson's r** | **P-value** | **Correlation Strength** |
| --- | --- | --- | --- |
| 2D-PCstandard vs. 2D-PCMVAAo | 0.413 | 0.040 | Moderate |
| 2D-PCstandard vs. 2D-PCMVdirect | 0.798 | <0.001 | Strong |
| 2D-PCstandard vs. Volumetric | 0.126 | 0.540 | Weak |
| 2D-PCstandard vs. 4D-flowstandard | 0.836 | <0.001 | Strong |
| 2D-PCstandard vs. 4D-flowMVAAo | 0.075 | 0.790 | Weak |
| 2D-PCstandard vs. 4D-flowMVdirect | 0.661 | 0.007 | Moderate |
| 2D-PCMVAAo vs. 2D-PCMVdirect | 0.360 | 0.077 | Weak |
| 2D-PCMVAAo vs. Volumetric | -0.129 | 0.539 | Weak |
| 2D-PCMVAAo vs. 4D-flowstandard | 0.671 | 0.006 | Moderate |
| 2D-PCMVAAo vs. 4D-flowMVAAo | 0.310 | 0.261 | Weak |
| 2D-PCMVAAo vs. 4D-flowMVdirect | 0.699 | 0.004 | Moderate |
| 2D-PCMVdirect vs. Volumetric | 0.351 | 0.085 | Weak |
| 2D-PCMVdirect vs. 4D-flowstandard | 0.751 | 0.001 | Strong |
| 2D-PCMVdirect vs. 4D-flowMVAAo | 0.230 | 0.409 | Weak |
| 2D-PCMVdirect vs. 4D-flowMVdirect | 0.639 | 0.010 | Moderate |
| Volumetric vs. 4D-flowstandard | 0.314 | 0.255 | Weak |
| Volumetric vs. 4D-flowMVAAo | 0.364 | 0.183 | Weak |
| Volumetric vs. 4D-flowMVdirect | 0.255 | 0.360 | Weak |
| 4D-flowstandard vs. 4D-flowMVAAo | 0.320 | 0.246 | Weak |
| 4D-flowstandard vs. 4D-flowMVdirect | 0.523 | 0.046 | Moderate |
| 4D-flowMVAAo vs. 4D-flowMVdirect | 0.212 | 0.449 | Weak |

*Correlation strength classification: r ≥ 0.9: excellent; r = 0.7-0.89: strong; r = 0.4-0.69: moderate; r < 0.4: weak*

## Figure S0. Heatmap of correlation coefficients and p-values for MVR volume measurements across various CMR flow quantification methods.

The correlation matrix includes data exclusively from the 15 patients who underwent both 2D-PC and 4D-flow acquisition. Pearson correlation coefficients (r) are displayed between pairs of methods, with a color scale ranging from -1 (negative correlation, blue) to 1 (positive correlation, red). P-values are annotated on the heatmap. (Correlation classifications: r ≥ 0.9, excellent correlation; r = 0.7–0.89, strong correlation; r = 0.4–0.69, moderate correlation; r = 0.1–0.39, weak correlation).

**
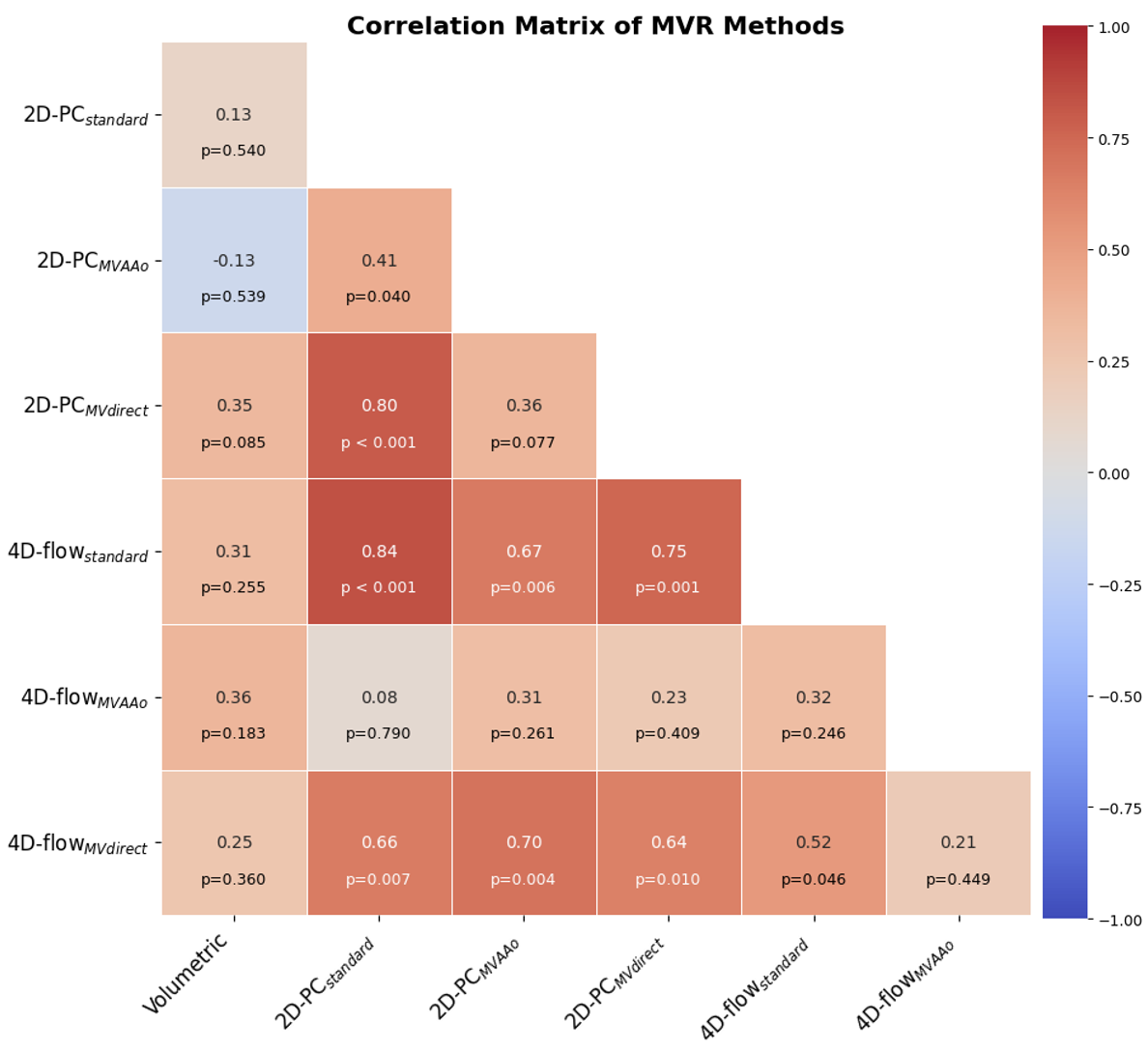
**

**Scatter Plots for Pearson's Correlation**

The following scatter plots show the relationships between different MVR quantification methods. These correlation plots correspond to the Bland-Altman plots presented in Figure 5 of the main manuscript.

Figure S2: Scatter plots showing correlations between different methods for quantifying mitral valve regurgitation. Each plot shows the linear relationship between two methods with the correlation coefficient (r) and p-value indicated.


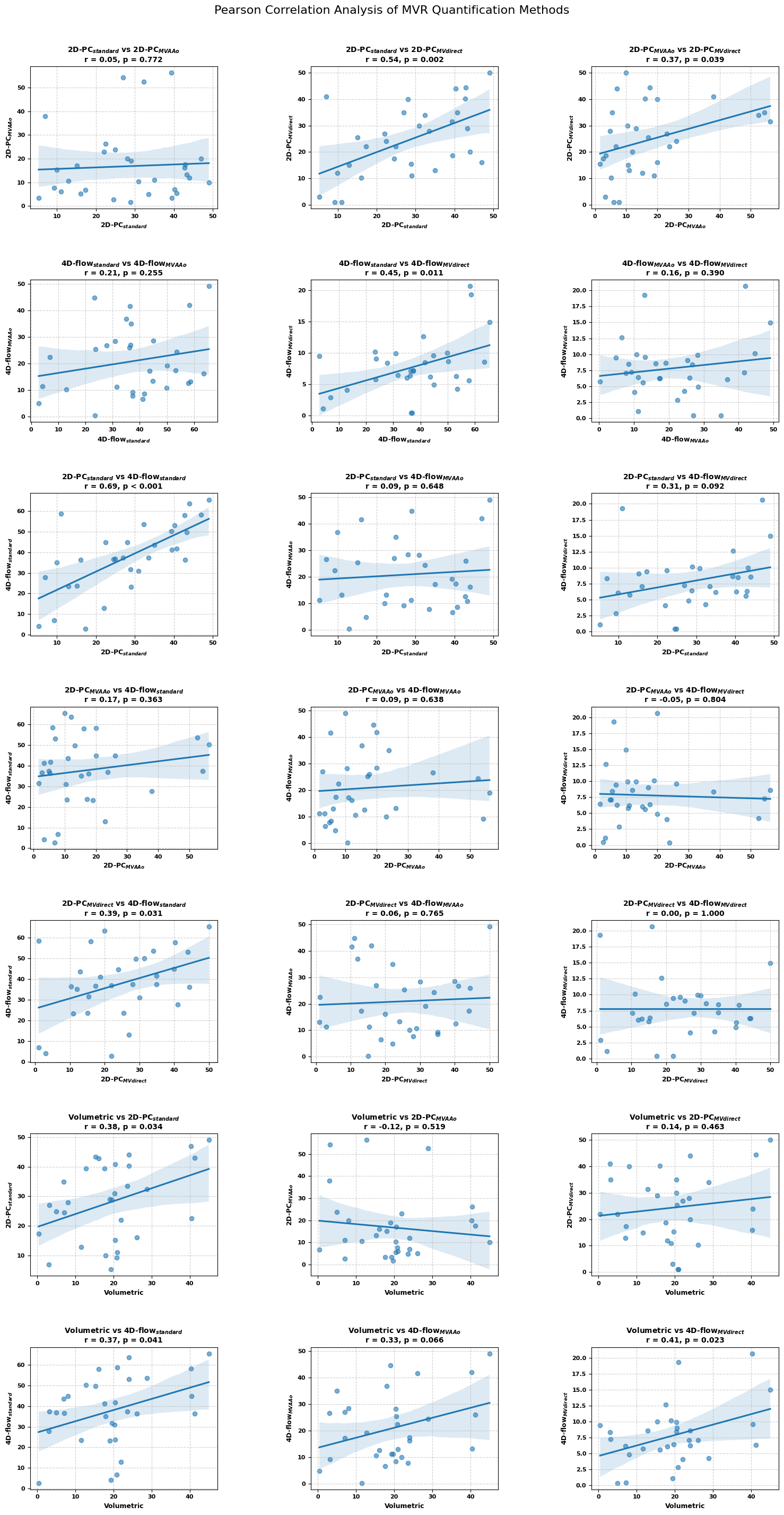


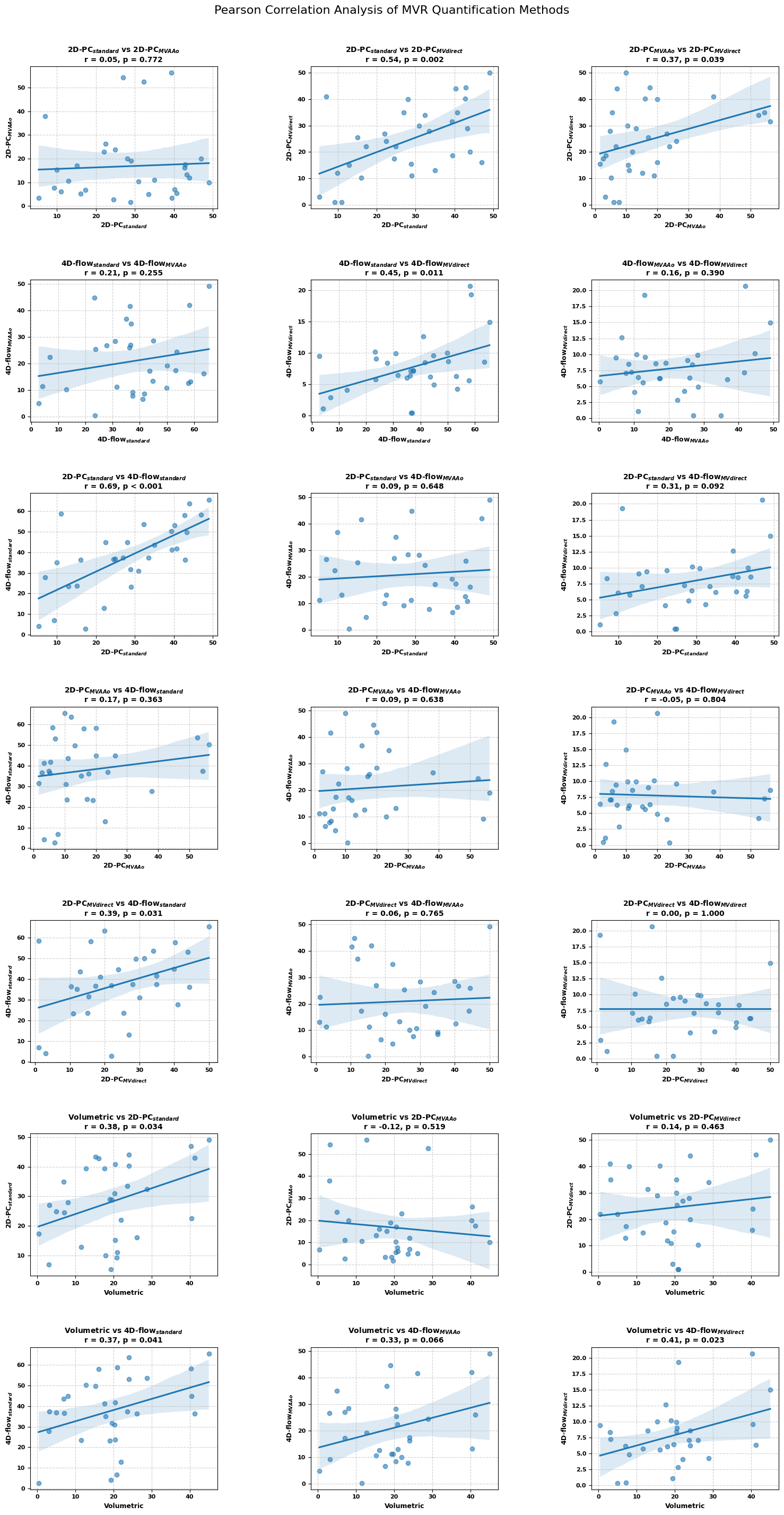

Supplement: Supplementary file 1 — Supplementary Material 1 [file 10554_2025_3421_MOESM1_ESM.docx]
